# Supplementary material for: COVID the Catalyst for Evolving Professional Role Identity? A Scoping Review of Global Pharmacists’ Roles and Services as a Response to the COVID-19 Pandemic
Source: Pharmacy (Basel). 2021 May 4;9(2):99. doi: 10.3390/pharmacy9020099 (PMC8162558; doi:10.3390/pharmacy9020099)
Supplement: Supplementary file 1 [file pharmacy-09-00099-s001.zip › pharmacy-1196704 - Table S4_change to S5.docx]

**Table S5.** Top 25 ranked roles and services performed by pharmacists during COVID-19.

| **Rank** | **Subcategory Roles/Services *[Definition]*** | **Number of references** |
| --- | --- | --- |
| 1 | **Interprofessional Collaboration**  *Encompasses activities related to teamwork and communication between members of the same profession and across members of different healthcare professions.* | 44 |
| 2 | **Patient Education**  *Involves providing education to patients regarding their disease management, COVID-19 treatments, or overall health.* | 41 |
| 3 | **Provider Education**  *Corresponds to education or information provided to other clinicians regarding COVID-19 treatments, current evidence, etc.* | 32 |
| 4 | **Guidance and Policy Development**  *Refers to developing material for the purposes of guiding therapy decisions and ensuring appropriate use of available (e.g., developing guidance documents, institutional guidelines, order sets, treatment protocols, and drug formularies).* | 25 |
| 5 | **Remote Patient Counseling**  *Includes counseling patients remotely (i.e., not in the same room) via telephone, video consult, message platforms, etc.* | 22 |
| 6 | **Redeployed/Backfilling Other Services**  *Refers to references of pharmacists being reassigned or redeployed to other service areas (e.g., field hospitals, COVID wards) and backfilling other services.* | 21 |
|  | **Monitoring/Follow-Up**  *Pharmacists provided monitoring services and follow-ups with patients.* | 21 |
| 7 | **Drug Stewardship**  *Pharmacists providing careful and responsible management of drug utilization (e.g., minimizing waste, antimicrobial stewardship)* | 19 |
|  | **Clinical Trials Management**  *Includes assessing patient’s clinical trial eligibility, activities related to clinical trials and clinical research, and investigational drug services (e.g., staffing investigational drug pharmacies, facilitating study activations at new sites, and ensuring investigational drug supply).* | 19 |
| 8 | **Evaluating Evidence**  *Pharmacists’ role in evaluating the available and current evidence on COVID-19 treatments and public health measures to inform colleagues and patients.* | 18 |
|  | **Rounding on COVID Patients**  *Pharmacists participated in medical rounds for COVID-19 patients and provided their clinical judgement and includes remote and in-person rounding.* | 18 |
|  | **Remote Patient Care/Home Monitoring**  *Using technology to facilitate monitoring of patients or providing care remotely in the patient’s home without them needing to attend the hospital, clinic, or pharmacy (e.g., virtual wellness checks).* | 18 |
| 9 | **Remote Collaboration**  *Pharmacists provided remote or virtual consultations for colleagues (e.g., physicians, nurses).* | 16 |
|  | **COVID Screening, Reporting, or Testing**  *Includes performing a patient assessment to determine the likelihood that a patient has contracted the COVID-19 virus and subsequently referring them to the authorities or signposting them to relevant services. It also includes performing COVID-19 testing by means of a validated testing device.* | 16 |
| 10 | **Managing Drug Shortages**  *Includes all roles related to drug shortages because of the COVID-19 pandemic (e.g., rationing medicines, managing short-dated stock, monitoring shortages).* | 15 |
| 11 | **Public Health Messaging**  *Pharmacists provided public health advice beyond their pharmacy’s patients to the wider community and public (e.g., hand hygiene, physical distancing, staying home if unwell, etc.). It also included different platforms pharmacists used to deliver these public health messages (e.g., radio, TV, text messaging, signage, etc.)* | 14 |
| 12 | **Simplifying Medication Regimens/Adjusting Timing**  *Pharmacists’ roles in adjusting dose timing or frequency to reduce nurses’ exposure on COVID wards to administer medications (e.g., using once-daily dosing, all morning doses given at a specific time etc.)* | 13 |
|  | **Home Delivery**  *Delivering medications to the residences of patients.* | 13 |
|  | **Nursing Support**  *Encompasses activities that support the roles of nurses, such as being physically or remotely present to answer medication-related queries, anticipating medication administration needs, and managing medication supply on the unit.* | 13 |
| 13 | **Prescribing, deprescribing, emergency refills**  *Includes pharmacist prescribing, deprescribing, extending prescriptions, providing emergency refills, repeat authorizations, legal dispensing of essential medications without a prescription, and/or activities surrounding relaxed legislation of controlled substances.* | 12 |
|  | **Modifying Medication Dispensing Processes**  *Includes changes made to pharmacy usual dispensing processes, for example, online ordering/prescriptions for physicians, online refill services for patients, coordinating home iv infusion service, etc.* | 12 |
|  | **Compounding**  *Involves preparing custom formulations for the purposes of conserving medication supply or meeting demand. Examples include compounding alcohol-based hand sanitizer and centrally preparing intravenous mixtures or infusions into larger sizes or higher concentrations.* | 12 |
| 14 | **Adjusting Medications due to Shortages, Therapeutic Substitutions**  *Pharmacists’ roles that include prescribing or recommending changes to medications due to drug shortages and providing appropriate dose equivalency information: for example, switching within a drug class or across therapeutic groups.* | 11 |
|  | **Transition of Care**  *Includes all pharmacist activities and services related to transition of patients to an alternate level of care, such as from tertiary to primary care. Examples include discharge medication reconciliation, coordinating supply of discharge medications, and following up with patients’ post-discharge.* | 11 |
| 15 | **Technology Support**  *Includes activities related to supporting patients or clinicians in the utilization of information technology. It includes support during video consultations or use of other electronic services as well as providing decision support for clinicians, medication and order set builds, managing alerts, and updating pump libraries.* | 9 |
