# Supplementary material for: COVID the Catalyst for Evolving Professional Role Identity? A Scoping Review of Global Pharmacists’ Roles and Services as a Response to the COVID-19 Pandemic
Source: Pharmacy (Basel). 2021 May 4;9(2):99. doi: 10.3390/pharmacy9020099 (PMC8162558; doi:10.3390/pharmacy9020099)
Supplement: Supplementary file 1 [file pharmacy-09-00099-s001.zip › pharmacy-1196704 -Table S1_change to S3.docx]

| Table S3 - Included Articles References (in alphabetical order) |
| --- |
| 1. Abdallah I, Eltahir A, Fernyhough L, El-Bardissy A, Ahmed R, Abdulgelil M, et al. The experience of Hamad General Hospital collaborative anticoagulation clinic in Qatar during the COVID-19 pandemic. J. Thromb. Thrombolysis 2020. 2. Aburas W, Alshammari TM. Pharmacists' roles in emergency and disasters: COVID-19 as an example. Saudi Pharm J 2020;28(12):1797-816. 3. Ahmad A, Alkharfy KM, Alrabiah Z, Alhossan A. Saudi Arabia, pharmacists and COVID-19 pandemic. J Pharm Policy Pract. 2020;13:41. 4. Ahuja T, Merchan C, Arnouk S, Cirrone F, Dabestani A, Papadopoulos J. COVID-19 pandemic preparedness: A practical guide from clinical pharmacists' perspective. Am. J. Health Syst. Pharm. 2020;77(18):1510-5. 5. Alexander M, Jupp J, Chazan G, O'Connor S, Chan A. Global oncology pharmacy response to COVID-19 pandemic: Medication access and safety. J. Oncol. Pharm. Pract. 2020;26(5):1225-9. 6. Alves da Costa F, Lee V, Leite SN, Murillo MD, Menge T, Antoniou S. Pharmacists reinventing their roles to effectively respond to COVID-19: a global report from the international pharmacists for anticoagulation care taskforce (iPACT). J Pharm Policy Pract 2020;13:12. 7. Arain S, Thalapparambath R, Al Ghamdi FH. COVID-19 pandemic: Response plan by the Johns Hopkins Aramco Healthcare inpatient pharmacy department. Res. Social Adm. Pharm. 2021;17(1):2009-11. 8. Atif M, Malik I. COVID-19 and community pharmacy services in Pakistan: challenges, barriers and solution for progress. J Pharm Policy Pract. 2020;13:33. 9. Bhat S, Farraye FA, Moss AC. Impact of Clinical Pharmacists in Inflammatory Bowel Disease Centers During the COVID-19 Pandemic. Am J Gastroenterol. 2020;115(9):1532-3. 10. Bragazzi NL, Mansour M, Bonsignore A, Ciliberti R. The Role of Hospital and Community Pharmacists in the Management of COVID-19: Towards an Expanded Definition of the Roles, Responsibilities, and Duties of the Pharmacist. Pharmacy (Basel) 2020;8(3). 11. Burgess LH, Cooper MK, Wiggins EH, Miller KM, Murray E, Harris S, et al. Utilizing Pharmacists to Optimize Medication Management Strategies During the COVID-19 Pandemic. J. Pharm. Pract. 2020:897190020961655. 12. Cheong MWL. 'To be or not to be in the ward': The Impact of Covid-19 on the Role of Hospital-Based Clinical Pharmacists - A Qualitative Study. J Am Coll Clin Pharm 2020. 13. Collins CD, West N, Sudekum DM, Hecht JP. Perspectives from the frontline: A pharmacy department's response to the COVID-19 pandemic. Am. J. Health Syst. Pharm. 2020;77(17):1409-16. 14. Como M, Carter CW, Larose-Pierre M, O'Dare K, Hall CR, Mobley J, et al. Pharmacist-Led Chronic Care Management for Medically Underserved Rural Populations in Florida During the COVID-19 Pandemic. Prev. Chronic Dis. 2020;17:E74. 15. de Val J, Sohal G, Sarwar A, Ahmed H, Singh I, Coleman JJ. Investigating the challenges and opportunities for medicines management in an NHS field hospital during the COVID-19 pandemic. Eur J Hosp Pharm. 2020. 16. DeRemer CE, Reiter J, Olson J. Transitioning ambulatory care pharmacy services to telemedicine while maintaining multidisciplinary collaborations. Am. J. Health Syst. Pharm. 2021;78(5):371-5. 17. Dzierba AL, Pedone T, Patel MK, Ciolek A, Mehta M, Berger K, et al. Rethinking the Drug Distribution and Medication Management Model: How a New York City Hospital Pharmacy Department Responded to COVID-19. J Am Coll Clin Pharm 2020. 18. Elbeddini A, Botross A, Gerochi R, Gazarin M, Elshahawi A. Pharmacy response to COVID-19: lessons learnt from Canada. J Pharm Policy Pract 2020;13(1):76. 19. Elbeddini A, Prabaharan T, Almasalkhi S, Tran C. Pharmacists and COVID-19. J Pharm Policy Pract 2020;13:36. 20. Erstad BL. Caring for the COVID Patient: A Clinical Pharmacist's Perspective. Ann Pharmacother. 2020:1060028020954224. 21. Ferguson NC, Quinn NJ, Khalique S, Sinnett M, Eisen L, Goriacko P. Clinical Pharmacists: An Invaluable Part of the Coronavirus Disease 2019 Frontline Response. Crit. care explor. 2020;2(10):e0243. 22. Garcia-Gil M, Velayos-Amo C. Hospital Pharmacist experience in the Intensive Care Unit: Plan COVID. Farm Hosp.. 2020;44(7):32-5. 23. Goff DA, Ashiru-Oredope D, Cairns KA, Eljaaly K, Gauthier TP, Langford BJ, et al. Global contributions of pharmacists during the COVID-19 pandemic. J Am Coll Clin Pharm 2020;3:1480-92. 24. Herzik KA, Bethishou L. The impact of COVID-19 on pharmacy transitions of care services. Res. Social Adm. Pharm.2021;17(1):1908-12. 25. Hua X, Gu M, Zeng F, Hu H, Zhou T, Zhang Y, et al. Pharmacy administration and pharmaceutical care practice in a module hospital during the COVID-19 epidemic. J. Am. Pharm. Assoc.2020;60(3):431-8.e. 26. Hussain K, Ambreen G, Muzammil M, Raza SS, Ali U. Pharmacy services during COVID-19 pandemic: experience from a tertiary care teaching hospital in Pakistan. J Pharm Policy Pract 2020;13(1):74. 27. Ibrahim OM, Ibrahim RM, Abdel-Qader DH, Al Meslamani AZ, Al Mazrouei N. Evaluation of Telepharmacy Services in Light of COVID-19. Telemed J. E Health 2020. 28. Ibrahim OM, Ibrahim RM, Z Al Meslamani A, Al Mazrouei N. Role of telepharmacy in pharmacist counselling to coronavirus disease 2019 patients and medication dispensing errors. J. Telemed. Telecare 2020:1357633X20964347. 29. Kasahun GG, Kahsay GM, Asayehegn AT, Demoz GT, Desta DM, Gebretekle GB. Pharmacy preparedness and response for the prevention and control of coronavirus disease (COVID-19) in Aksum, Ethiopia; a qualitative exploration. BMC Health Serv. Res. 2020;20(1):913. 30. Koster ES, Philbert D, Bouvy ML. Impact of the COVID-19 epidemic on the provision of pharmaceutical care in community pharmacies. Res. Social Adm. Pharm. 2021;17(1):2002-4. 31. Kristina SA, Herliana N, Hanifah S. The perception of role and responsibilities during covid-19 pandemic: A survey from indonesian pharmacists. International Journal of Pharmaceutical Research 2020;12(Supplementry 2):3034-9. 32. Lemtiri J, Matusik E, Cousein E, Lambiotte F, Elbeki N. The role of the critical care pharmacist during the COVID-19 pandemic. Ann. Pharm. Fr. 2020;78(6):464-8. 33. Li H, Zheng S, Liu F, Liu W, Zhao R. Fighting against COVID-19: Innovative strategies for clinical pharmacists. Res. Social Adm. Pharm. 2020. 34. Li M, Razaki H, Mui V, Rao P, Brocavich S. The pivotal role of pharmacists during the 2019 coronavirus pandemic J. Am. Pharm. Assoc.2020;60(6):e73-e5. 35. Liao Y, Ma C, Lau AH, Zhong M. Role of pharmacists during the COVID-19 pandemic in China - Shanghai Experiences. J Am Coll Clin Pharm 2020. 36. Lim RHM, Shalhoub R, Sridharan BK. The experiences of the community pharmacy team in supporting people with dementia and family carers with medication management during the COVID-19 pandemic. Res. Social Adm. Pharm. 2021;17(1):1825-31. 37. Lin CH, Lin YW, Wang JY, Lin MH. The pharmaceutical practice of mask distribution by pharmacists in Taiwan's community pharmacies under the Mask Real-Name System, in response to the COVID-19 outbreak. Cost Eff. Resour. Alloc. 2020;18:45. 38. Liu S, Luo P, Tang M, Hu Q, Polidoro JP, Sun S, et al. Providing pharmacy services during the coronavirus pandemic. Int. J. Clin. Pharm. 2020;42(2):299-304. 39. Margusino-Framinan L, Illarro-Uranga A, Lorenzo-Lorenzo K, Monte-Boquet E, Marquez-Saavedra E, Fernandez-Bargiela N, et al. Pharmaceutical care to hospital outpatients during the COVID-19 pandemic. Telepharmacy. Farm Hosp. 2020;44(7):61-5. 40. McConachie S, Martirosov D, Wang B, Desai N, Jarjosa S, Hsaiky L. Surviving the surge: Evaluation of early impact of COVID-19 on inpatient pharmacy services at a community teaching hospital. Am. J. Health Syst. Pharm. 2020;77(23):1994-2002. 41. Meng L, Qiu F, Sun S. Providing pharmacy services at cabin hospitals at the coronavirus epicenter in China. Int. J. Clin. Pharm. 2020;42(2):305-8. 42. Merchan C, Soliman J, Ahuja T, Arnouk S, Keeley K, Tracy J, et al. COVID-19 pandemic preparedness: A practical guide from an operational pharmacy perspective. Am. J. Health Syst. Pharm. 2020;77(19):1598-605. 43. Merks P, Jakubowska M, Drelich E, Świeczkowski D, Bogusz J, Bilmin K, et al. The legal extension of the role of pharmacists in light of the COVID-19 global pandemic. Res. Social Adm. Pharm. 2021;17(1):1807-12. 44. Mohammad I, Berlie HD, Lipari M, Martirosov AL, Duong AA, Faraj M, et al. Ambulatory Care Practice in the COVID-19 Era: Redesigning Clinical Services and Experiential Learning. J Am Coll Clin Pharm. 2020;3:1129-37. 45. Morgan R. COVID-19 disaster response: A pharmacist volunteer's experience at the epicenter. Am. J. Health Syst. Pharm. 2020;77(21):1786-8. 46. Mukattash TL, Jarab AS, Mukattash I, Nusair MB, Farha RA, Bisharat M, et al. Pharmacists' perception of their role during COVID-19: a qualitative content analysis of posts on Facebook pharmacy groups in Jordan. Pharm. Pract. (Granada) 2020;18(3):1900. 47. Nguy J, Hitchen SA, Hort AL, Huynh C, Rawlins MDM. The role of a Coronavirus disease 2019 pharmacist: an Australian perspective. Int. J. Clin. Pharm. 2020;42(5):1379-84. 48. Ou HT, Yang YHK. Community Pharmacists in Taiwan at the Frontline Against the Novel Coronavirus Pandemic: Gatekeepers for the Rationing of Personal Protective Equipment. Ann. Intern. Med. 2020;173(2):149-50. 49. Parreiras Martins MA, Fonseca de Medeiros A, Dias Carneiro de Almeida C, Moreira Reis AM. Preparedness of pharmacists to respond to the emergency of the COVID-19 pandemic in Brazil: a comprehensive overview. Drugs Ther Perspect 2020:1-8. 50. Paudyal V, Cadogan C, Fialova D, Henman MC, Hazen A, Okuyan B, et al. Provision of clinical pharmacy services during the COVID-19 pandemic: Experiences of pharmacists from 16 European countries. Res. Social Adm. Pharm. 2020. 51. Pelaez Bejarano A, Villar Santos P, Robustillo-Cortes MdLA, Sanchez Gomez E, Santos Rubio MD. Implementation of a novel home delivery service during pandemic. Eur J Hosp Pharm 2020. 52. Peris-Marti JF, Bravo-Jose P, Saez-Lleo C, Fernandez-Villalba E. Specialized pharmaceutical care in social health centers in the times of COVID-19. Farm Hosp. 2020;44(7):43-8. 53. Schiller DS, Fulman M, Champagne J, Awad N. COVID-19 pandemic planning, response, and lessons learned at a community hospital. Am. J. Health Syst. Pharm. 2020. 54. Segal EM, Alwan L, Pitney C, Taketa C, Indorf A, Held L, et al. Establishing clinical pharmacist telehealth services during the COVID-19 pandemic. Am. J. Health Syst. Pharm. 2020;77(17):1403-8. 55. Shen B, Chen L, Zhang L, Zhang M, Li J, Wu J, et al. Wuchang Fangcang Shelter Hospital: Practices, Experiences, and Lessons Learned in Controlling COVID-19. SN comprehensive clinical medicine 2020:1-6. 56. Surapat B, Sungkanuparph S, Kirdlarp S, Lekpittaya N, Chunnguleum K. Role of clinical pharmacists in telemonitoring for patients with Coronavirus Disease 2019 (COVID-19). J. Clin. Pharm. Ther. 2020. 57. Truong L, Whitfield K, Nickerson-Troy J, Francoforte K. Drive-thru anticoagulation clinic: Can we supersize your care today? J. Am. Pharm. Assoc.2020. 58. Ung COL. Community pharmacist in public health emergencies: Quick to action against the coronavirus 2019-nCoV outbreak. Res. Social Adm. Pharm. 2020;16(4):583-6. 59. Visacri MB, Figueiredo IV, Lima TdM. Role of pharmacist during the COVID-19 pandemic: A scoping review. Res. Social Adm. Pharm. 2020. 60. Wallis N, Gust C, Porter E, Gilchrist N, Amaral A. Implementation of field hospital pharmacy services during the COVID-19 pandemic. Am. J. Health Syst. Pharm. 2020. 61. Yi ZM, Hu Y, Wang GR, Zhao RS. Mapping Evidence of Pharmacy Services for COVID-19 in China. Front. Pharmacol. 2020;11:555753. 62. Ying W, Qian Y, Kun Z. Drugs supply and pharmaceutical care management practices at a designated hospital during the COVID-19 epidemic. Res. Social Adm. Pharm. 2021;17(1):1978-83. 63. Zuckerman AD, Patel PC, Sullivan M, Potts A, Knostman M, Humphreys E, et al. From natural disaster to pandemic: A health-system pharmacy rises to the challenge. Am. J. Health Syst. Pharm. 2020. |

Supplementary Table S1.
