# Supplementary material for: COVID the Catalyst for Evolving Professional Role Identity? A Scoping Review of Global Pharmacists’ Roles and Services as a Response to the COVID-19 Pandemic
Source: Pharmacy (Basel). 2021 May 4;9(2):99. doi: 10.3390/pharmacy9020099 (PMC8162558; doi:10.3390/pharmacy9020099)
Supplement: Supplementary file 1 [file pharmacy-09-00099-s001.zip › pharmacy-1196704 -Table S5_change to S2.docx]

**Table S2.** Appendix: Search Strategies.

| **Database** | **Search Strategy** |
| --- | --- |
| **MEDLINE**  **Ovid MEDLINE(R) ALL** 1946 to December 11, 2020 | 1. (pharmacist* or pharmacy or pharmacies or druggist*).mp.  2. pharmaceutic* service*.mp.  3. exp Pharmaceutical Services/  4. pharmaceutical care.mp.  5. exp Medication Therapy Management/  6. ((medication or medicine* or drug* or prescription* or dose* or dosage*) adj3 manag*).mp.  7. or/1-6  8. limit 7 to covid-19 |
| **Embase**  **Ovid Embase** 1974 to 2020 December 11 | 1. (pharmacist* or pharmacy or pharmacies or druggist*).mp.  2. pharmaceutic* service*.mp.  3. exp "pharmacy (shop)"/  4. pharmaceutical care.mp.  5. exp medication therapy management/  6. ((medication or medicine* or drug* or prescription* or dose* or dosage*) adj3 manag*).mp.  7. or/1-6  8. limit 7 to covid-19 |
| **CINAHL** | S1 pharmacist* or pharmacy or pharmacies or druggist*  S2 "pharmaceutic* service*"  S3 "pharmaceutical care"  S4 (MH "Medication Management")  S5 (medication N2 manag*) or (medicine* N2 manag*) or (drug* N2 manag*) or (prescription* N2 manag*) or (dose* N2 manag*) or (dosage* N2 manag*)  S6 S1 OR S2 OR S3 OR S4 OR S5  S7 (MH "Coronavirus+") OR (MH "Coronavirus Infections+")  S8 coronavirus* or corona virus* or OC43 or NL63 or 229E or HKU1 or HCoV* or ncov* or covid* or sars-cov* or sarscov* or Sars-coronavirus* or "Severe Acute Respiratory Syndrome Coronavirus*"  S9 S7 OR S8  S10 SARS or SARS-CoV or MERS or MERS-CoV or Middle East respiratory syndrome or camel* or dromedar* or equine or coronary or coronal or covidence* or covidien or influenza virus or HIV or bovine or calves or TGEV or feline or porcine or BCoV or PED or PEDV or PDCoV or FIPV or FCoV or SADS-CoV or canine or CCov or zoonotic or avian influenza or H1N1 or H5N1 or H5N6 or IBV or murine corona*  S11 S9 NOT S10  S12 pneumonia or covid* or coronavirus* or corona virus* or ncov* or 2019-ncov or sars*  S13 (MH "Pneumonia+") AND Wuhan  S14 (2019-ncov or ncov19 or ncov-19 or 2019-novel CoV or sars-cov2 or sars-cov-2 or sarscov2 or sarscov-2 or Sars-coronavirus2 or Sars-coronavirus-2 or SARS-like coronavirus* or coronavirus-19 or covid19 or covid-19 or covid 2019 or ((novel or new or nouveau) N2 (CoV or nCoV or covid or coronavirus* or corona virus or Pandemi*)) or ((covid or covid19 or covid-19) and pandemic*) or (coronavirus* and pneumonia)  S15 S11 OR S12 OR S13 OR S14  S16 DT 20191201-20300101  S17 S6 AND S15 AND S16 |
| **Scopus** | ( ( TITLE-ABS-KEY ( ( coronavirus*  OR  "corona virus*"  OR  oc43  OR  nl63  OR  229e  OR  hku1  OR  hcov*  OR  ncov*  OR  covid*  OR  "sars-cov*"  OR  sarscov*  OR  "Sars-coronavirus*"  OR  "Severe Acute Respiratory Syndrome Coronavirus*"  OR  d614g ) ) )  AND NOT  ( ( TITLE-ABS-KEY ( ( sars  OR  sars-cov  OR  mers  OR  mers-cov  OR  "Middle East respiratory syndrome"  OR  camel*  OR  dromedar*  OR  equine  OR  coronary  OR  coronal  OR  covidence*  OR  covidien  OR  "influenza virus"  OR  hiv  OR  bovine  OR  calves  OR  tgev  OR  feline  OR  porcine  OR  bcov ) ) )  OR  ( TITLE-ABS-KEY ( ( ped  OR  pedv  OR  pdcov  OR  fipv  OR  fcov  OR  sads-cov  OR  canine  OR  ccov  OR  zoonotic  OR  "avian influenza"  OR  h1n1  OR  h5n1  OR  h5n6  OR  ibv  OR  "murine corona*" ) ) ) ) )  OR  ( TITLE-ABS-KEY ( ( pneumonia  OR  covid*  OR  coronavirus*  OR  "corona virus*"  OR  ncov*  OR  2019-ncov  OR  sars* )  AND  wuhan )  OR  ( ( 2019-ncov  OR  ncov19  OR  ncov-19  OR  2019-novel  AND  cov  OR  sars-cov2  OR  sars-cov-2  OR  sarscov2  OR  sarscov-2  OR  sars-coronavirus2  OR  sars-coronavirus-2  OR  "SARS-like coronavirus*"  OR  coronavirus-19  OR  covid19  OR  covid-19  OR  "covid 2019"  OR  ( ( covid  OR  covid19  OR  covid-19 )  AND  pandemic* )  OR  ( coronavirus*  AND  pneumonia ) ) ) )  OR  ( TITLE ( ( novel  OR  new  OR  nouveau )  AND  ( cov  OR  ncov  OR  covid  OR  coronavirus*  OR  "corona virus"  OR  pandemi* ) ) )  OR  ( ABS ( ( novel  OR  new  OR  nouveau )  AND  ( cov  OR  ncov  OR  covid  OR  coronavirus*  OR  "corona virus"  OR  pandemi* ) ) )  OR  ( KEY ( ( novel  OR  new  OR  nouveau )  AND  ( cov  OR  ncov  OR  covid  OR  coronavirus*  OR  "corona virus"  OR  pandemi* ) ) )  AND  TITLE-ABS-KEY ( pharmacist*  OR  pharmacy  OR  pharmacies  OR  druggist* OR "pharmaceutic* service*" OR "pharmaceutical care" OR (medication W/2 manag*) or (medicine* W/2 manag*) or (drug* W/2 manag*) or (prescription* W/2 manag*) or (dose* W/2 manag*) or (dosage* N2 manag*) )  AND  ( LIMIT-TO ( PUBYEAR ,  2021 )  OR  LIMIT-TO ( PUBYEAR ,  2020 )  OR  LIMIT-TO ( PUBYEAR ,  2019 ) ) |
| **Web of Science Core Collection** | **#1** TS= ( ( coronavirus* OR "corona virus*" OR oc43 OR nl63 OR 229e OR hku1 OR hcov* OR ncov* OR covid* OR "sars-cov*" OR sarscov* OR "Sars-coronavirus*" OR "Severe Acute Respiratory Syndrome Coronavirus*" OR d614g ) NOT ( sars OR sars-cov OR mers OR mers-cov OR "Middle East respiratory syndrome" OR camel* OR dromedar* OR equine OR coronary OR coronal OR covidence* OR covidien OR "influenza virus" OR hiv OR bovine OR calves OR tgev OR feline OR porcine OR bcov OR ped OR pedv OR pdcov OR fipv OR fcov OR sads-cov OR canine OR ccov OR zoonotic OR "avian influenza" OR h1n1 OR h5n1 OR h5n6 OR ibv OR "murine corona*" ))  **#2** TS=( ( ( pneumonia OR covid* OR coronavirus* OR "corona virus*" OR ncov* OR 2019-ncov OR sars* ) AND wuhan ) OR ( 2019-ncov OR ncov19 OR ncov-19 OR 2019-novel AND cov OR sars-cov2 OR sars-cov-2 OR sarscov2 OR sarscov-2 OR sars-coronavirus2 OR sars-coronavirus-2 OR "SARS-like coronavirus*" OR coronavirus-19 OR covid19 OR covid-19 OR "covid 2019" ) OR ( ( covid OR covid19 OR covid-19 ) AND pandemic*) OR ( coronavirus* AND pneumonia ) )  **#3** TI= ( ( novel OR new OR nouveau ) AND ( cov OR ncov OR covid OR coronavirus* OR "corona virus" OR pandemi* ) )  **#4** AB= ( ( novel OR new OR nouveau ) AND ( cov OR ncov OR covid OR coronavirus* OR "corona virus" OR pandemi* ) )  **#5** TS= ( ( novel OR new OR nouveau ) AND ( cov OR ncov OR covid OR coronavirus* OR "corona virus" OR pandemi* ) )  **#6** #1 OR #2 OR #3 OR #4 OR #5  **#7** TS= ( pharmacist* OR pharmacy OR pharmacies OR druggist* OR "pharmaceutic* service*" OR "pharmaceutical care" OR (medication NEAR/2 manag*) OR (medicine* NEAR/2 manag*) OR (drug* NEAR/2 manag*) OR (prescription* NEAR/2 manag*) OR (dose* NEAR/2 manag*) OR (dosage* NEAR/2 manag*) )  **#8** #6 AND #7 [Filter by date: 2019 - 2020] |
| **Google Scholar** | (pharmacists OR pharmacy) AND covid-19 |
